# Supplementary material for: Explaining the increment in coronary heart disease mortality in Mexico between 2000 and 2012
Source: PLoS One. 2020 Dec 3;15(12):e0242930. doi: 10.1371/journal.pone.0242930 (PMC7714134; doi:10.1371/journal.pone.0242930)
Supplement: S1 Appendix — (DOCX) [file pone.0242930.s001.docx]

**S1 Appendix: Main data sources for the parameters used in the Mexican IMPACT Model for 2000 to 2012**

| **Variables** |  | **2002** | **2012** |
| --- | --- | --- | --- |
| Population |  | National Population Council (CONAPO) | National Population Council (CONAPO) |
| Deaths |  | National System of Health Information of the Ministry of Health (SINAIS) | National System of Health Information of the Ministry of Health (SINAIS) |
|  |  |  |  |
| **Risk factors** | |  |  |
| Current smoking | | National Health Survey (ENSA) 2000 | National Nutrition and Health Survey (ENSANut) 2012 |
| Systolic blood pressure | | National Health Survey (ENSA) 2000 | National Nutrition and Health Survey (ENSANut) 2012 |
| Cholesterol |  | Global Burden of Metabolic Risk Factors of Chronic Diseases Collaborating Group | Global Burden of Metabolic Risk Factors of Chronic Diseases Collaborating Group |
| Physical activity | | National Health Survey (ENSA) 2000 | National Nutrition and Health Survey (ENSANut) 2012 |
| Obesity (BMI) | | National Health Survey (ENSA) 2000 | National Nutrition and Health Survey (ENSANut) 2012 |
| Diabetes |  | National Health Survey (ENSA) 2000 | National Nutrition and Health Survey (ENSANut) 2012 |
|  |  |  |  |
| **Patient numbers** | |  |  |
| Number of MI patients | | Hospital Discharges at SINAIS | Hospital Discharges at SINAIS |
| Number of unstable angina pectoris patients | | Hospital Discharges at SINAIS | Hospital Discharges at SINAIS |
| CABG |  | Hospital Discharges at SINAIS | Hospital Discharges at SINAIS |
| PTCA |  | Hospital Discharges at SINAIS | Hospital Discharges at SINAIS |
| Medication |  | Hospital Discharges at SINAIS | Hospital Discharges at SINAIS |
| Secondary prevention following MI | | National Registry of Acute Coronary Syndromes (RENASICA) II | National Registry of Acute Coronary Syndromes (RENASICA) III |
| Secondary prevention following CABG/ PTCA | | National Registry of Acute Coronary Syndromes (RENASICA) II | National Registry of Acute Coronary Syndromes (RENASICA) III |
| Congestive heart failure patients | | National Health Survey (ENSA) 2000 | National Nutrition and Health Survey (ENSANut) 2012 |
|  | Treatments | Expert panel | Expert panel |
| Chronic angina patients | | National Health Survey (ENSA) 2000 | National Nutrition and Health Survey (ENSANut) 2012 |
|  | Treatments | Expert panel | Expert panel |
| Antihypertensive medication | | National Health Survey (ENSA) 2000 | National Nutrition and Health Survey (ENSANut) 2012 |
| Statins for primary prevention | | National Health Survey (ENSA) 2000 | National Nutrition and Health Survey (ENSANut) 2012 |

Datasets from the Mexican Ministry of Health, Mexican National Population Council (CONAPO) and National Nutrition and Health Surveys are publicly available and anonymous. In the case National Registry of Acute Coronary Syndromes, the data are available upon request from the RENASICA Executive Committee
