# Supplementary material for: Explaining the increment in coronary heart disease mortality in Mexico between 2000 and 2012
Source: PLoS One. 2020 Dec 3;15(12):e0242930. doi: 10.1371/journal.pone.0242930 (PMC7714134; doi:10.1371/journal.pone.0242930)
Supplement: S2 Appendix — (DOCX) [file pone.0242930.s002.docx]

# **S2 Appendix:**

# **Clinical efficacy of interventions: relative risk reductions obtained from meta-analyses, and randomised controlled trials***

| **TREATMENTS** | | **Relative Risk Reduction**  (95% CI) | **Comments** | **Source paper:**  **First author (year), notes** |
| --- | --- | --- | --- | --- |
| **Acute myocardial infarction** | | |  |  |
| Thrombolysis | 31%  (95% CI: 14, 45) | | <55 yrs: OR=0.692; RRR=30.8 (95% CI: 14-45)  55-64 yrs: OR=0.736; RRR=26.4 (95% CI: 17-40)  65-74 yrs: OR=0.752; RRR=24.8 (95% CI: 15-37)  >75 yrs: OR=0.844; RRR=15.6 (95% CI: 4-30) | Estess(2002)^[[1]](#endnote-1)^, [updated FTT] |
| Aspirin | 15%  (95% CI: 11, 19) | | OR=0.85 (95% CI: 0.81, 0.89). RRR 15% (95% CI: 11,19)  page 75:outcome is vascular and nonvascular deaths | Antithrombotic  Trialists' Collaboration (2002)^[[2]](#endnote-2)^ |
| Primary angioplasty STEMI | 32%  (95% CI: 5, 50) | | OR 0.68 (95% CI: 0.50, 0.95). RRR 32% (95% CI: 5,50)  outcome compares primary angioplasty to thrombolytics, not specific to STEMI, in results on page 3. | Cucherat (2003).^[[3]](#endnote-3)^ |
| Primary PTCA Non- STEMI | 32%  (95% CI: 5, 51) | | OR 0.65 (95% CI: 0.49, 0.95). RRR 32% (95% CI: 5,51) for  cardiovascular death on page 917. [RRR for cardiovascular death or MI was 26 (95% CI: 3,44) and was 24 (95% CI: 0,42)  for any death] | RITA 3 (Fox 2005)^[[4]](#endnote-4)^ |
| Primary CABG surgery | 39%  (95% CI: 23, 52) | | OR 0.61 (95% CI: 0.48, 0.77). RRR 39% (95% CI: 23,52) on  page 565, 0-5 yr mortality | Yusuf (1994)^[[5]](#endnote-5)^ |
| Beta blockers | 4%  (95% CI: -8, 15) | | OR 0.96 (95% CI: 0.85, 1.08), RR 4% (95% CI: -8,15) on page 1732. | Freemantle (1999)^[[6]](#endnote-6)^ |
| ACE inhibitors | 7%  (95% CI: 2, 11) | | OR 0.93, (0.89, 0.98), RR 7% (2,11) for 30 day mortality in MI. | ACE Inhibitor Myocardial Infarction Collaborative Group 1998.^[[7]](#endnote-7)^ |

| **Cardio-pulmonary resuscitation**  **(CPR)** | |  |  |
| --- | --- | --- | --- |
| Community CPR USA | 5%  (95% CI: 4, 15.3) | Nichol study reports overall median survival to discharge at 7.4% in this multi-country/site review, page 520  The Model focuses on 30/7 survival. Discharge survival will therefore provide an over-estimate, which we have explicitly addressed by assuming 5% at 30/7.  Rea looks at odds of bystander dispatcher assisted CPR and bystander CPR without dispatch assistance and compares to No bystander CPR. 7265 out-of-hospital arrests attended. OR  0.59 - 0.69 for these two groups which would give RRRs of 41% and 31%. [Consider as crude equivalent of CPR to no CPR comparison]. 15.3% survival to discharge in King- county, WA; consider as maximum value. Use Nichol (1999)^28^ 5% as USA average.  Graham et al 1999 meta anlysis of papers 1973 - 1996 report 6.4% at discharge. Assume better in 2000, thus 6.4% at 30/7 OPALS RCT reports only 5.2%. | Nichol (1999)^[[8]](#endnote-8)^  Rea (2001)^[[9]](#endnote-9)^ |
| Hospital CPR USA | 33%  (95% CI: 10, 36) | AMI accounted for 35% of adult total cases. Adult survival to discharge 36% post VF or VT (majority of post AMI cases, only 10.6% post Asystole,  Adult survival to discharge 18% overall, but this reflected  ALL Medical arrests in hospital. (varied from 10-36% depending on type of initial rhythm) (tables 4 &5 page 55) Review of 36,000 adults with cardiac arrests in the 253 US/Canadian Hospitals National Registry of CPR. Nadkarni, JAMA, 2006:295 (1) 50-57)  Older article from Tunstall-Pedoe on page 1350 shows survival at 24 hrs to be 32%, discharge to home at 21%, and 1 year survival to be 15% overall. (16% and 8% in general wards, 31% and 16% in coronary care unit (page 1349), etc. Assume in USA 2000 is better.  Corroboration: Model assumes that approximately 2% AMI admissions have primary VF (Olmsted County study). This is consistent with NHDS discharge code of CPR in 0.74%  (2000), suggesting approximately 1/3 survive. | Nadkarni (2006)^[[10]](#endnote-10)^  NHDS discharge codes (2000) Tunstall-Pedoe(1992)^[[11]](#endnote-11)^ |
| Aspirin | 15%  (95% CI: 11, 19) | OR 0.85 (95% CI: 0.49, 0.95), RR 15% (95% CI: 11, 19)  outcome is vascular and nonvascular deaths on page 75. | Antithrombotic  Trialists' Collaboration (2002)^5^ |
| Beta blockers | 23% (95% CI: 15,31) | OR 0.77 (95% CI: 0.85, 0.69), 23% (95% CI: 15,31) | Freemantle (1999)^9^ |
| ACE inhibitors | 20% (95% CI: 13, 26( | OR 0.80 (95% CI: 0.74, 0.87), 20% (95% CI: 13,26) on page 1577, death up to 4 years (endpoint of study looking at those with heart failure or LV dysfunction) | Flather (2000)^[[12]](#endnote-12)^ |
| Statins | 22%  (95% CI: 10, 26) | OR=0.78 (95% CI: 0.74—0.84). RRR=22% (95% CI: 10, 26) RR=0.77 (95% CI: 0.68—0.87). RRR=23% (95% CI: 13,30) | Cholesterol Treatment Trialists’ Collaborators (2005)^[[13]](#endnote-13)^ |
|  |  | OR=0.77 (95% CI: 0.71-0.83). RRR=23% (95% CI: 17, 29)  Wilt (2004) Section CHD mortality, page 1430. | Wilt (2004)^[[14]](#endnote-14)^ |
| Warfarin | 22%  (95% CI: 13, 31) | OR=0.78 (95% CI: 0.67-0.90), RRR=22% (95% CI: 10, 33)  Meta-analysis looking at oral anticoagulant therapy in coronary artery disease (31 trials about 18000 patinets) by intensity of INR control: High intensity (INR>2.8) warfarin vs. control for outcome of death had OR of 0.78(95% CI: 0.69-0.87) corresponding to a RRR of 22% (95% CI: 13, 31); of Moderate intensity warfarin (INR 2-3.0) vs. control had OR of 0.82 (95% CI: 0.23-2.33) not significant but corresponding RR of 18% (95% CI: -133, 77) | Anand and Yusuf (1999)^[[15]](#endnote-15)^  Lau (1992)^[[16]](#endnote-16)^. Table 1, page 253 (anticoagulants) |
| Rehabilitation | 26% (95% CI: 10, 39) | OR= 0.74 (95% CI: 0.61-0.90), RRR = 26% (95% CI: 10, 39) in Fig 1, page 685 Taylor reference. | Taylor (2004)^[[17]](#endnote-17)^ |

| ***Chronic Angina*** | |  |  |
| --- | --- | --- | --- |
| CABG surgery years | 39% (95% CI: 23,52) | OR= 0.61 (95% CI: 0.48-0.77), RR 39% (95% CI: 23,52) on page 565, 5 yr mortality | Yusuf (1994)**^¡^** |
| 0-5 |  |  |  |
| CABG surgery years | 32% | OR= 0.83 (95% CI: 0.70-0.98), RR 17 (95% CI: 2,30) on | Yusuf (1994)**^¡^** |
| 6-10 | (95% CI: 2, 30) | page 565, 10 yr mortality OR= 0.68 (95% CI: 0.56-0.83), RR 32 (95% CI: 17,44) on page 565, 7 yr mortality CABG compared to medical treatment |  |
|  |  |  |  |
|  |  |  |  |
|  |  |  |  |
| Angioplasty inchronic angina, with stents | 13%  (95% CI: 0, 16) | OR=0.87 (95% CI: 0.52-1.45), RRR=13% (95% CI: -45, 48) | BASKET RCT (Lancet 2005  366, 921)^[[18]](#endnote-18)^: Comparison of drug |
|  |  |  | eluting stents vs. bare metal |
|  |  |  | stents  Folland (1997)^[[19]](#endnote-19)^ Table 3, all |
|  |  |  | deaths, 60 months follow up. |
|  |  | Maximum benefit, assume equivalent to CABG surgery for | Yusuf (1994)^25^ |
|  |  | two vessel disease CABG, OR 0.84, (RR 16% 2, 30) 5 year | Pocock (1995)^[[20]](#endnote-20)^: No difference |
|  |  | survival 88% in controls. | between PTCA and CABG as |
|  |  | Minimum assumption: NIL benefit | initial revasc procedure. |
|  |  |  | Ditto Bucher (2000)^[[21]](#endnote-21)^ |
| Aspirin | 15% | OR= 0.85 (95% CI: 0.81-0.89), RR 15% (95% CI: 11,19) | Antithrombotic |

| Statins | **22%**  **(**95% CI: 10-26) | RR=0.78 (95% CI: 0.74—0.84). RRR=22% (95% CI: 10, 26)  RR=0.77 (95% CI: 0.68—0.87). RRR=23% (95% CI: 13,30) | Cholesterol Treatment Trialists’ Collaborators (2005)^[[22]](#endnote-22)^ |  |
| --- | --- | --- | --- | --- |
|  |  | in those with other CHD |  |  |
| ***Unstable Angina*** |  |  |  |  |
| Aspirin alone | 15% | OR= 0.85 (95% CI: 0.81-0.89), RR 15% (95% CI: 11,19)  outcome is vascular and nonvascular deaths on page 75. Assume appropriate for unstable angina patients | Antithrombotic |  |
|  | | (95% CI: 11, 19) | OR= 0.85 (95% CI: 0.81-0.89), RR 15% (95% CI: 11,19) Trialists' Collaboration (2002)^12^  outcome is vascular and nonvascular deaths on page 75. Assume appropriate for unstable angina patients  OR 0.67 (95% CI: 0.48,1.02) RR 33% (95% CI: -2, 56) in |  |
| Aspirin & Heparin | 33% | OR= 0.85 (95% CI: 0.81-0.89), RR 15% (95% CI: 11,19)  outcome is vascular and nonvascular deaths on page 75. Assume appropriate for unstable angina patients  OR 0.67 (95% CI: 0.48,1.02) RR 33% (95% CI: -2, 56) in  table 2. The study outcome is composite MI death and non | Oler (1996)^[[23]](#endnote-23)^ |  |
|  | (95% CI: -2,56) |  | . |  |
|  |  | fatal MI, compares those on ASA+Hep to ASA only |  |  |
| Platelet glycoprotein | 9% | RR 0.91 (95% CI: 0.84, 0.98) RR 9% (95% CI: 2,16) study | Boersma (2002)^[[24]](#endnote-24)^ |  |
| IIB/IIIA inhibitors | (95% CI: 2,16) | looked at acute coronary syndrome without persistent ST |  |  |
|  |  | elevation |  |  |
| Primary PTCA Non- | 32% | OR 0.68 (95% CI: 0.49, 0.95). RRR 32% (95% CI: 5, 51) for | RITA 3 (Fox 2005)^24^ |  |
| STEMI | (95% CI: 5-51) |  | Cardiovascular deaths, table 3 |  |
| Primary CABG | **43%** | OR 0.57 (95% CI: 0.40, 0.81). RR 43% (95% CI: 19,60) | Yusuf (1994)^25^ |  |
| surgery | (95% CI: 19,60) | reduction in mortality at 5 years in those with class III/IV |  |  |
|  |  | angina, table 4, page 566. |  |  |

| ***Heart failure in patients requiring hospitalisation*** | |  |  |
| --- | --- | --- | --- |
| ACE inhibitors | 20% (95% CI:  13,26 | OR 0.80 (95% CI: 0.74, 0.87). RR 20% (95% CI: 13,26) on  page 1577, [death up to 4 years was study endpoint for those with heart failure or LV dysfunction]. | Flather (2000)^32^ |
| Beta blockers | 35% (95% CI:  26,43) | OR 0.65 (95% CI: 0.57, 0.74). RR 35% (95% CI: 26,43) : all cause mortality | Shibata (2001)^44^ |
| Spironolactone | 30% | OR 0.70 (95% CI: 0.59, 0.82). RR 30% (95% CI: 18,41) in | Pitt (1999)^45^ |
|  | (95% CI: 18, 41) | those that had at least one cardiac related hospitalization. [ |  |
|  |  | 31% (95% CI: 18-42) in entire study population of those with |  |
|  |  | CHF, page 711 ] |  |
| Aspirin | 15% | OR= 0.85 (95% CI: 0.81, 0.89), RR 15% (95% CI: 11,19) | Antithrombotic |
|  | (95% CI: 11,19) | outcome is vascular and nonvascular deaths on page 75. | Trialists' Collaboration (2002)^12^ |
| Statins | 22%  (95% CI: 10-26%) | OR=0.78 (95% CI: 0.74, 0.84). RRR=22% (95% CI: 10-26),  post AMI | Cholesterol Treatment Trialists’ Collaborators (2005)^33^ |
|  |  | OR=0.77 (95% CI: 0.68, 0.87). RRR=23% (95% CI: 13,30) in |  |
|  |  | those with other CHD |  |

| ***Heart failure in the community*** | |  |  |
| --- | --- | --- | --- |
| ACE inhibitors |  | OR 0.80 (95% CI: 0.74, 0.87). RR 20% (95% CI: 13,26) on | Flather (2000)^32^ |
|  | 20% | page 1577, death up to 4 years [in those with heart failure or |  |
|  | (95% CI: 13,26) | LV dysfunction]. |  |
| Beta blockers | 35%  (95% CI: 26,43) | OR 0.65 (95% CI: 0.57, 0.74). RR 35 (95% CI: 26,43). Section 3.3 page 353 | Shibata (2001)^[[25]](#endnote-25)^ |
| Spironolactone | 31% | OR 0.69 (95% CI: 0.58, 0.82). RR 31% (95% CI: 18-42) in | Pitt (1999)^[[26]](#endnote-26)^ |
|  | (95% CI: 18, 42) | entire study population consisting of those with CHF, page |  |
|  |  | 711 [30 (95% CI: 18, 41) in those with a cardiac related |  |
|  |  | hospitalization]. |  |
| Aspirin | 15%  (95% CI: 11, 19) | OR= 0.85 (0.81, 0.89), RR 15% (11,19) outcome is vascular and nonvascular deaths on page 75. Assume appropriate for | Antithrombotic  Trialists' Collaboration (2002)5 |
|  |  | patients with CHF due to CHD |  |
| Statins | 22%  (95% CI: 10-26%) | OR=0.78 (95% CI: 0.74, 0.84). RRR=22% (95% CI: 10-26)  OR=0.77 (95% CI: 0.68, 0.87). RRR=23% (95% CI: 13,30) in | Cholesterol Treatment Trialists’ Collaborators (2005)8 |
|  |  | those with other CHD |  |

| ***Hypertension treatment*** | |  |  |
| --- | --- | --- | --- |
|  | 13%  (95% CI: 6,19) | OR 0.87 (95% CI: 0.81, 0.94). RRR 13% (95% CI: 6, 19) in  those with high blood pressure without disease at entry. [RRR 29% (95% CI: 17, 37) those with average blood  pressure and CHD, treated with ACEI] | Law (2003)^[[27]](#endnote-27)^ |
|  |  |  |  |
| ***Therapies for primary prevention of raised cholesterol*** | |  |  |
| Statins | 35%  (95% CI: 11, 52) | OR 0.65 (95% CI: 0.48, 0.89). 35% (95% CI: 11,52) for CHD  mortality (only trials using statins), figure 3 on page 4 | Pignone (2000)^[[28]](#endnote-28)^ |
| Gemfibrozil | 7%  (95% CI: -8, 19) | OR 0.93 (95% CI: 0.81, 1.08); RRR 7% (95% CI: -8, 19) | Studer (2005)^[[29]](#endnote-29)^ |
| Niacin | 5%  (95% CI: -10, 18) | OR 0.95 (95% CI: 0.82, 1.10); RRR 5% (95% CI: -10, 0.18) | Studer (2005)^32^ |

***** Relative Risk Reduction calculated as 1- Odds Ratio

26

# **Age-specific case fatality rates for each patient group**

| **GROUP** | **AMI** | **Post AMI** | **Unstable**  **Angina** | **CABG**  **surgery** | **Angioplasty** | **Heart**  ***Hospital*** | **Failure**  ***Community*** | **HypertensionHypercholesteraemia** | |  |
| --- | --- | --- | --- | --- | --- | --- | --- | --- | --- | --- |
| *Interval* | **30 day** | **One year*** | **One year*** | **One year*** | **One year*** | **One year** | **One year** | **One year** | **One year** |  |
| **Mean** | 0.084 | 0.051 | 0.069 | 0.020 | 0.016 | 0.246 | 0.081 | 0.010 | 0.006 |  |
| **MEN 25-34** | *0.011* | 0.008 | 0.016 | 0.003 | 0.003 | 0.034 | 0.011 | 0.000 | 0.000 |  |
| **35-44** | *0.012* | 0.009 | 0.024 | 0.005 | 0.005 | 0.068 | 0.022 | 0.001 | 0.001 |  |
| **45-54** | *0.023* | 0.017 | 0.034 | 0.007 | 0.007 | 0.096 | 0.032 | 0.002 | 0.002 |  |
| **55-64** | *0.054* | 0.034 | 0.056 | 0.012 | 0.012 | 0.140 | 0.045 | 0.006 | 0.006 |  |
| **65-74** | *0.101* | *0.073* | 0.070 | 0.023 | 0.025 | 0.283 | 0.093 | 0.014 | 0.014 |  |
| **75-84** | *0.164* | *0.122* | 0.091 | 0.042 | 0.042 | 0.337 | 0.111 | 0.035 | 0.035 |  |
| **85+** | *0.279* | *0.189* | 0.118 | 0.075 | 0.074 | 0.418 | 0.138 | 0.094 | 0.094 |  |
| **WOMEN 25-34** | *0.011* | *0.004* | *0.016* | *0.003* | *0.003* | 0.034 | 0.011 | 0.000 | 0.000 |  |
| **35-44** | *0.013* | *0.006* | *0.024* | *0.005* | *0.005* | 0.068 | 0.022 | 0.001 | 0.001 |  |
| **45-54** | *0.026* | *0.010* | *0.034* | *0.007* | *0.007* | 0.096 | 0.032 | 0.001 | 0.001 |  |
| **55-64** | *0.061* | *0.019* | *0.056* | *0.012* | *0.012* | 0.140 | 0.045 | 0.002 | 0.002 |  |
| **65-74** | *0.114* | *0.084* | *0.070* | *0.023* | *0.027* | 0.222 | 0.081 | 0.007 | 0.007 |  |
| **75-84** | *0.167* | *0.116* | *0.091* | *0.042* | *0.039* | 0.289 | 0.094 | 0.021 | 0.021 |  |
| **85+** | *0.267* | *0.177* | *0.118* | *0.075* | *0.061* | 0.368 | 0.121 | 0.079 | 0.079 |  |
| ***SOURCE*** | **Medicare^2^** | **Medicare^2^** | **Van Domberg^[[30]](#endnote-30)^** | **Medicare^2^** | **Medicare^2^** | **Medicare^2^** | **Medicare^2^** | **NHANES** | **& Vital Statistics^2^** |  |

*excluding heart failure patients (already considered within heart failure groups)

1. Estess JM, Topol EJ. Fibrinolytic treatment for elderly patients with acute myocardial infarction. Heart 2002;87:308-11. [↑](#endnote-ref-1)
2. Antithrombotic Trialists' Collaboration. Collaborative meta-analysis of randomised trials of antiplatelet therapy for prevention of death, myocardial infarction, and stroke in high risk patients. BMJ 2002;324:71-86. [↑](#endnote-ref-2)
3. Cucherat M, Bonnefoy E, Tremeau G. Primary angioplasty versus intravenous thrombolysis for acute myocardial infarction. Cochrane Database Syst Rev 2000;CD001560. [↑](#endnote-ref-3)
4. Fox KA, Poole-Wilson P, Clayton TC et al. 5-year outcome of an interventional strategy in

   non-ST-elevation acute coronary syndrome: the British Heart Foundation RITA 3 randomised trial. Lancet 2005;366:914-20. [↑](#endnote-ref-4)
5. Yusuf S, Zucker D, Peduzzi P et al. Effect of coronary artery bypass graft surgery on survival: overview of 10-year results from randomised trials by the Coronary Artery Bypass Graft Surgery Trialists Collaboration. Lancet 1994;344:563-70. [↑](#endnote-ref-5)
6. Freemantle N, Cleland J, Young P, Mason J, Harrison J. beta Blockade after myocardial infarction: systematic review and meta regression analysis. BMJ 1999;318:1730-7. [↑](#endnote-ref-6)
7. ACE Inhibitor Myocardial Infarction Collaborative Group. Indications for ACE inhibitors in the early treatment of acute myocardial infarction: systematic overview of individual data from 100,000 patients in randomized trials. ACE Inhibitor Myocardial Infarction Collaborative Group. Circulation 1998;97:2202-12. [↑](#endnote-ref-7)
8. Nichol G, Stiell IG, Hebert P, Wells GA, Vandemheen K, Laupacis A. What is the quality of life for survivors of cardiac arrest? A prospective study. Acad Emerg Med 1999;6:95-102. [↑](#endnote-ref-8)
9. Rea TD, Eisenberg MS, Culley LL, Becker L. Dispatcher-assisted cardiopulmonary resuscitation and survival in cardiac arrest. Circulation 2001;%20;104:2513-6. [↑](#endnote-ref-9)
10. Nadkarni VM, Larkin GL, Peberdy MA et al. First documented rhythm and clinical outcome from in-hospital cardiac arrest among children and adults. JAMA 2006;295:50-7. [↑](#endnote-ref-10)
11. # Tunstall-Pedoe H, Bailey L, Chamberlain DA, et al. Survey of 3765 cardiopulmonary resuscitations in British hospitals (the BRESUS Study): methods and overall results. BMJ 1992;304:1347-51.

    [↑](#endnote-ref-11)
12. Flather MD, Yusuf S, Kober L et al. Long-term ACE-inhibitor therapy in patients with heart failure or left- ventricular dysfunction: a systematic overview of data from individual patients. ACE-Inhibitor Myocardial Infarction Collaborative Group. Lancet 2000;355:1575-81. [↑](#endnote-ref-12)
13. Baigent C, Keech A, Kearney PM et al. Efficacy and safety of cholesterol-lowering treatment: prospective meta-analysis of data from 90,056 participants in 14 randomised trials of statins. Lancet 2005;366:1267-78. [↑](#endnote-ref-13)
14. Wilt TJ, Bloomfield HE, MacDonald R et al. Effectiveness of statin therapy in adults with coronary heart disease. Arch Intern Med 2004;164:1427-36. [↑](#endnote-ref-14)
15. Anand SS, Yusuf S. Oral anticoagulant therapy in patients with coronary artery disease: a meta-analysis. JAMA 1999;282:2058-67. [↑](#endnote-ref-15)
16. Lau J, Antman EM, Jimenez-Silva J, Kupelnick B, Mosteller F, Chalmers TC. Cumulative meta-analysis of therapeutic trials for myocardial infarction. N Engl J Med 1992;327:248-54. [↑](#endnote-ref-16)
17. Taylor RS, Brown A, Ebrahim S et al. Exercise-based rehabilitation for patients with coronary heart disease: systematic review and meta-analysis of randomized controlled trials. Am J Med 2004;116:682-92. [↑](#endnote-ref-17)
18. Kaiser C, Brunner-La Rocca HP, Buser PT et al. Incremental cost-effectiveness of drug-eluting stents compared with a third-generation bare-metal stent in a real-world setting: randomised Basel Stent Kosten Effektivitats Trial (BASKET). Lancet 2005;366:921-9. [↑](#endnote-ref-18)
19. Folland ED, Hartigan PM, Parisi AF. Percutaneous transluminal coronary angioplasty versus medical therapy for stable angina pectoris: outcomes for patients with double-vessel versus single-vessel coronary artery disease in a Veterans Affairs Cooperative randomized trial. Veterans Affairs ACME Investigators. J Am Coll Cardiol 1997;29:1505-11. [↑](#endnote-ref-19)
20. Pocock SJ, Henderson RA, Rickards AF et al. Meta-analysis of randomised trials comparing coronary angioplasty with bypass surgery. Lancet 1995;346:1184-9. [↑](#endnote-ref-20)
21. Bucher HC, Hengstler P, Schindler C, Guyatt GH. Percutaneous transluminal coronary angioplasty versus medical treatment for non-acute coronary heart disease: meta-analysis of randomised controlled trials. BMJ 2000;321:73-7. [↑](#endnote-ref-21)
22. Yusuf S, Zucker D, Peduzzi P et al. Effect of coronary artery bypass graft surgery on survival: overview of 10-year results from randomised trials by the Coronary Artery Bypass Graft Surgery Trialists Collaboration. Lancet 1994;344:563-70. [↑](#endnote-ref-22)
23. Oler A, Whooley MA, Oler J, Grady D. Adding heparin to aspirin reduces the incidence of myocardial infarction and death in patients with unstable angina. A meta-analysis. JAMA 1996;276:811-5. [↑](#endnote-ref-23)
24. Boersma E, Harrington RA, Moliterno DJ et al. Platelet glycoprotein IIb/IIIa inhibitors in acute coronary syndromes: a meta-analysis of all major randomised clinical trials. Lancet 2002;359:189-98. [↑](#endnote-ref-24)
25. Shibata MC, Flather MD, Wang D. Systematic review of the impact of beta blockers on mortality and hospital admissions in heart failure. Eur J Heart Fail 2001;3:351-7. [↑](#endnote-ref-25)
26. # Pitt B, Zannad F, Remme WJ et al. The effect of spironolactone on morbidity and mortality in patients with severe heart failure. Randomized Aldactone Evaluation Study Investigators. N Engl J Med 1999;341:709-17.

    [↑](#endnote-ref-26)
27. Law M, Wald N, Morris J. Lowering blood pressure to prevent myocardial infarction and stroke: a new preventive strategy. Health Technol Assess 2003;7:1-94. [↑](#endnote-ref-27)
28. Pignone M, Phillips C, Mulrow C. Use of lipid lowering drugs for primary prevention of coronary heart disease: meta-analysis of randomised trials. BMJ 2000;321:983-6. [↑](#endnote-ref-28)
29. Studer M, Briel M, Leimenstoll B, Glass TR, Bucher HC. Effect of different antilipidemic agents and diets on mortality: a systematic review. Arch Intern Med 2005;165:725-30. [↑](#endnote-ref-29)
30. van Domburg RT, Miltenburg-van-Zijl AJ, Veerhoek RJ, Simoons ML. Unstable angina: good long-term outcome after a complicated early course. J Am Coll Cardiol 1998;31:1534-9. [↑](#endnote-ref-30)
