# Supplementary material for: Explaining the increment in coronary heart disease mortality in Mexico between 2000 and 2012
Source: PLoS One. 2020 Dec 3;15(12):e0242930. doi: 10.1371/journal.pone.0242930 (PMC7714134; doi:10.1371/journal.pone.0242930)
Supplement: S3 Appendix — (DOCX) [file pone.0242930.s003.docx]

**S3 Appendix:**

# **MAIN ASSUMPTIONS AND OVERLAP ADJUSTMENTS USED IN THE MEXICAN IMPACT MODEL**

| **Treatment category** | **Assuptions and overlap adjustments** | | **Justification** |
| --- | --- | --- | --- |
| Post_AMI patients | Assume 25% already counted as HF patients | | UNAL 2004 |
|  |  | Therefore assume residual case fatality halved, h |  |
|  |  | having transferred these HF patients to the HF groups |  |
| Post-CABG patients | Assume 2/3 had MI, already counted as Post AMI | | UNAL 2004 |
| Post- PTCA survivor | Assume 50% had prior AMI, already counted as Post AMI | | UNAL 2004 |
|  |  | Assume 25% also had CABG thus already counted as post CABG | NHDS |
|  |  | Assume 25% had prior PTCA, so already counted | NHDS |
| Chronic angina treatment | Assume that 20% of PTCA go to CABG | | NHDS |
| Angina in the community | Stat with the total patient number with angina in the community | | Capewell 2000 |
|  |  | Then deduct patients counted elsewhere: |  |
|  |  | - Patients already treated for unstable angina in hospital |  |
|  |  | - 50% of those receiving CABG for angina |  |
|  |  | -50% of those receivin secondary prevention post AMI |  |
| Heart failure in the community | Based on NHANES prevalence | | NHANES |
|  |  | Assume 50% of heart failure is due to CHD |  |
|  |  | Deduct patients treated for severe heart failure the hospital |  |
| Fall in popuation blood pressure | Estimate the number of DPPs by hypertension treatment | | Capewell 1999 |
|  |  | Then substract this from the total DPPs attributed to the secular fall in population BP | Capewell 2000 |
|  |  |  |  |

Additional assumptions:

1. We assumed that adherence, or the proportion of treated patients actually taking therapeutically effective levels of medication, was 100% among hospitalized patients, 70% among symptomatic patients in the community, and 50% among asymptomatic patients in the community [29, 30].
2. In the case of cholesterol levels in Mexican population, we only obtained information of the mean by sex in the population and used the same value for every age group.
3. For physical activity, we only obtained information from 2206-2012, and used a linear estimation to obtain the values for 2000.
