# Supplementary material for: Explaining the increment in coronary heart disease mortality in Mexico between 2000 and 2012
Source: PLoS One. 2020 Dec 3;15(12):e0242930. doi: 10.1371/journal.pone.0242930 (PMC7714134; doi:10.1371/journal.pone.0242930)
Supplement: S4 Appendix. Specific beta coefficients or RR’s for major risk factors: Data sources, values and comments — (DOCX) [file pone.0242930.s004.docx]

# **S4 Appendix: Specific Beta Coefficients or RR’s for major risk factors: data sources, values and comments.**

Estimated β coefficients from multiple regression analyses for the relationship between absolute changes in population mean risk factors and % changes in coronary heart disease mortality for men and women, stratified by age.

Age groups (years)

SYSTOLIC BLOOD PRESSURE

| 25-44 | 45-54 | 55-64 | 65-74 | 75-84 |
| --- | --- | --- | --- | --- |
| **0.49** | **0.49** | **0.52** | **0.58** | **0.65** |
| **-0.036** | **-0.035** | **-0.032** | **-0.027** | **-0.021** |
| -0.029 | -0.028 | -0.026 | -0.022 | -0.017 |
| -0.043 | -0.042 | -0.039 | -0.032 | -0.025 |
| **0.40** | **0.40** | **0.49** | **0.52** | **0.59** |
| **-0.046** | **-0.046** | **-0.035** | **-0.032** | **-0.026** |
| -0.037 | -0.037 | -0.028 | -0.026 | -0.021 |
| -0.055 | -0.055 | -0.042 | -0.039 | -0.031 |

**Men** (hazard ratio per 20 mmHg)

**Men** (log hazard ratio per 1 mmHg)

Min Max

**Women** (hazard ratio per 20 mmHg)

**Women** (log hazard ratio per 1 mmHg)

Min Max

Source: Prospective studies collaborative meta-analysis, Lancet 2002 ^[[1]](#endnote-1)^

*UNITS: % mortality change per 20 mmHg change in Systolic BP

Strengths: massive dataset, includes US data, adjusted for regression dilution bias, consistent with randomized clinical trials, results stratified by sex and age, with 95% CIs

Limitations: some publication bias still possible.

**CHOLESTEROL** Age groups (years)

|  | 25-44 | 45-54 | 55-64 | 65-74 | 75-84 | 85+ |
| --- | --- | --- | --- | --- | --- | --- |
| **Men & Women** (Mortality |  |  |  |  |  |  |
| reduction per 1 mmol/l) | **0.900** | **0.650** | **0.450** | **0.333** | **0.317** | **0.250** |
| **Log coefficient** | **-1.2942** | **-0.8238** | **-0.5245** | **-0.3719** | **-0.3512** | **-0.2709** |
| Lower 95% CI | -1.035 | -0.659 | -0.420 | -0.298 | -0.281 | -0.217 |
| Upper 95% CI | -1.553 | -0.989 | -0.629 | -0.446 | -0.421 | -0.325 |

Source: Law & Wald meta-analysis

*UNITS: % mortality change per 1 mmol/l (38.6 mg/dl) change in total cholesterol

Strengths: includes US data, adjusted for regression dilution bias, includes randomized clinical trials, RCT values consistent with observational data, results stratified by sex and age, with 95% CIs

Limitations: some publication bias still possible.

**BODY MASS INDEX (BMI)**

Age groups (years)

|  | <44 | 45-59 | 60-69 | 70-79 | 80+ |
| --- | --- | --- | --- | --- | --- |
| Risk reduction per 1 kg/m^2^: James Asia |  |  |  |  |  |
| Pacific data | 0.1100 | 0.0900 | 0.0500 | 0.0400 | 0.0300 |
| Asia Pacific age gradient therefore: | 1.22 | **1.00** | 0.56 | 0.44 | 0.33 |
| Bogers relative risks, CHD deaths per 5 kg/m^2^ |  | **1.16** |  |  |  |
| Age specific relative risks per 1 kg/m^2^, |  |  |  |  |  |
| applying age gradients from James et al | 1.04 | **1.03** | 1.02 | 1.01 | 1.01 |
| **Men & Women, log coefficients*** | **0.0363** | **0.0297** | **0.0165** | **0.0132** | **0.0099** |
| Minimum values | 0.0255 | 0.0209 | 0.0116 | 0.0093 | 0.0070 |
| Maximum values *(from James et al)* | 0.0466 | 0.0381 | 0.0212 | 0.0169 | 0.0127 |

Source: Bogers et al.,^57^ James et al. 2004^58^

*UNITS: % mortality change per 1 kg/m^2^ change in BMI

Strengths: Large number of studies included. Adjusted for blood pressure, total cholesterol, and physical activity. 95% CIs also provided.

Limitations: Observational data; age gradient applied from James study.

# **Relative Risks for Smoking, Diabetes and Physical Inactivity for Coronary Heart Disease Mortality. (Best, Minimum and Maximum Estimates from the InterHeart Study)**

**Systolic blood pressure**

|  | **Age groups (years)** | | | | |
| --- | --- | --- | --- | --- | --- |
|  | **25-44** | **45-54** | **55-64** | **65-74** | **75-84** |
| **Men (HR per 20 mmHg)** | **0.49** | **0.49** | **0.52** | **0.58** | **0.65** |
| **Men (log HR per 1 mmHg)** | **-0.036** | **-0.035** | **-0.032** | **-0.027** | **-0.021** |
| Min | -0.029 | -0.028 | -0.026 | -0.022 | -0.017 |
| Max | -0.043 | -0.042 | -0.039 | -0.032 | -0.025 |
| **Women (HR per 20mmHg)** | **0.4** | **0.4** | **0.49** | **0.52** | **0.59** |
| **Women (log HR per 1 mmHg)** | **-0.046** | **-0.046** | **-0.035** | **-0.032** | **-0.026** |
| Min | -0.037 | -0.037 | -0.028 | -0.026 | -0.021 |
| Max | -0.055 | -0.055 | -0.042 | -0.039 | -0.031 |

Source: Prospective studies collaborative meta-analysis^[[2]](#endnote-2)^

*Measurement units: % change in mortality per 20 mmHg change in systolic blood pressure.

**Cholesterol**

|  | Age groups | | | | | |
| --- | --- | --- | --- | --- | --- | --- |
|  | 25-44 | 45-54 | 55-64 | 65-74 | 75-84 | 85+ |
| **Men and women** (Reduction per 1 mmol/l) | **0.9** | **0.65** | **0.45** | **0.333** | **0.317** | **0.25** |
| **Log coefficient** | **-1.2942** | **-0.8238** | **-0.5245** | **-0.3719** | **-0.3512** | **-0.2709** |
| Inferior 95% CI | -1.035 | -0.659 | -0.42 | -0.298 | -0.281 | -0.217 |
| Superior 95% CI | -1.553 | -0.989 | -0.629 | -0.446 | -0.421 | -0.325 |

Source: Law & Wald meta-analysis^[[3]](#endnote-3)^

*Measurement units: % mortality change per 1 mmol/l (38.6 mg/dl) in total cholesterol

**IMC**

| **IMC** | Age groups | | | | |
| --- | --- | --- | --- | --- | --- |
|  | <44 | 45-59 | 60-69 | 70-79 | 80+ |
| Risk reduction per 1 kg/m^2^ |  |  |  |  |  |
| James Asia | 0.11 | 0.09 | 0.05 | 0.04 | 0.03 |
| Gradient | 1.22 | **1** | 0.56 | 0.44 | 0.33 |
| Bogers RR |  |  |  |  |  |
| CHD deaths per 5 kg/m^2^ |  | **1.16** |  |  |  |
| Specific RR per age for 1 kg/m^2^, using James et.al. gradient | 1.04 | **1.03** | 1.02 | 1.01 | 1.01 |
| **log coefficient for men and women** | **0.0363** | **0.0297** | **0.0165** | **0.0132** | **0.0099** |
| Min | 0.0255 | 0.0209 | 0.0116 | 0.0093 | 0.007 |
| Max | 0.0466 | 0.0381 | 0.0212 | 0.0169 | 0.0127 |

Source: Bogers et al^[[4]](#endnote-4)^., James et al. 2004^[[5]](#endnote-5)^

*UNITS: % mortality change per 1 kg/m^2^ change in BMI

**Lifestiles INTERHEART study**

|  | **Both sexes** | | **Men** | | **Women** | |
| --- | --- | --- | --- | --- | --- | --- |
|  | **≤55 years** | **>55 years** | **≤55years** | **>55 years** | **≤65 years** | **>65 years** |
| Smoking | 3.33 (2.86-3.87) | 2.44 (2.10-2.84) | 3.33 (2.80-3.95) | 2.52 (2.15-2.96) | 4.49 (3.11-6.47) | 2.14 (1.35-3.39) |
| Physical activity | 0.95 (0.79-1.14) | 0.79 (0.66-0.94) | 1.02 (0.83-1.25)* | 0.79 (0.66-0.96) | 0.74 (0.49-0.87) | 0.75 (0.46-1.22) |
| Hypertension | 2.24 (1.93-2.60) | 1.72 (1.52-1.95) | 1.99 (1.66-2.39) | 1.72 (1.49-1.98) | 2.94 (2.25-3.85) | 1.82 (1.39-2.38) |
| Diabetes | 2.96 (2.40-3.64) | 2.05 (1.71-2.45) | 2.66 (2.04-3.46) | 1.93 (1.58-2.37) | 3.53 (2.49-5.01) | 2.59 (1.78-3.78) |

Source: Yusuf InterHEART Study. Lancet 2004.^[[6]](#endnote-6)^

1. Prospective Studies Collaboration. Body-mass index and cause-specific mortality in 900 000 adults: collaborative analyses of 57 prospective studies. The Lancet , Volume 373 , Issue 9669 , 1083 – 1096 [↑](#endnote-ref-1)
2. Law M, Wald N, Morris J. Lowering blood pressure to prevent myocardial infarction and stroke: a new preventive strategy. Health Technol Assess 2003;7:1-94. [↑](#endnote-ref-2)
3. Law MR, Wald NJ, Thompson SG. By how much and how quickly does reduction in serum cholesterol concentration lower risk of ischaemic heart disease? BMJ 1994;308:367-72. [↑](#endnote-ref-3)
4. Bogers RP, Hoogenveen RT, Boshuizen H. et al. Overweight and obesity increase the risk of coronary heart disease: a pooled analysis of 30 prospective studies. European Journal of Epidemiology 2006;21 (supplement):107 [↑](#endnote-ref-4)
5. Bogers RP, Hoogenveen RT, Boshuizen H. et al. Overweight and obesity increase the risk of coronary heart disease: a pooled analysis of 30 prospective studies. European Journal of Epidemiology 2006;21 (supplement):107 [↑](#endnote-ref-5)
6. Yusuf S, Hawken S, Ôunpuu S, et al. Effect of potentially modifiable risk factors associated with myocardial infarction in 52 countries (the INTERHEART study): case-control study. *The Lancet.* 2004;364(9438):937-952. [↑](#endnote-ref-6)
