# Supplementary material for: Explaining the increment in coronary heart disease mortality in Mexico between 2000 and 2012
Source: PLoS One. 2020 Dec 3;15(12):e0242930. doi: 10.1371/journal.pone.0242930 (PMC7714134; doi:10.1371/journal.pone.0242930)
Supplement: S5 Appendix — (DOCX) [file pone.0242930.s005.docx]

# **S5 Appendix: Uncertainty analysis: parameter distributions, functions and sources**

This table records the type of distribution and associated functions for each of the input variables in the IMPACT model. We implemented stochastic uncertainty analysis in Excel using Ersatz (version 1.0 available at <http://www.epigear.com>), an add-in that allows probabilistic bootstrapping in Excel . Ersatz allows repeated random draws from specified distributions for input variables that are used to recalculate iteratively the model. It then calculates the 95% uncertainty intervals from the realised values of the output variable (deaths prevented or postponed). For the IMPACT model, we calculated the uncertainty intervals based on 1000 draws taking the 95% uncertainty intervals as the 2.5^th^ and 97.5^th^ percentiles. Input variables taken from external sources (e.g. case fatality rates, beta coefficients and relative risk reductions) were randomly drawn from specified distributions.

| **Input parameters** | ***Type of distribution and functions (Mean, Standard error)*** | Source |
| --- | --- | --- |
| **Population** | | |
| Population counts and CHD deaths stratified by age, sex, and Index of Multiple Deprivation | - Population counts (no error) - Deaths expected in 2007 had CHD mortality rates in 2000 persisted (***Poisson distribution***) | Vital Statics |
| **Risk factors** | | |
| Prevalence/mean estimates (pooled data; national estimates for 1980 and 2012) | - Continuous variables (Body Mass Index, SBP, total cholesterol, fruit and vegetable consumption): (***Normal distribution***: mean, SE of mean) |  |
| Beta coefficient: **SBP** | ***Normal distribution*** (mean, SE of mean):  M < 45 (-0.036,0.004); M 45-54 (-0.035,0.004)  M 55-64 (-0.032,0.003); M 65-74 (-0.027,0.003)  M 75-84 (-0.021,0.002); M 85+ (-0.016,0.002)  F < 55 (-0.046, 0.005); F 55-64 (-0.035,0.004)  F 65-74 (-0.032,0.003); F 75-84 (-0.026,0.003)  F 85+ (-0.019,0.002) | Prospective studies collaborative meta-analysis (2002) [53]. Parameters on the log scale. |
| Beta coefficient: **total cholesterol** | ***Normal distribution*** (mean, SE of mean):  M < 45 (-0.799,0.081); M 45-54 (-0.755,0.077)  M 55-64 (-0.446,0.046); M 65-74 (-0.236,0.024)  M 75-84 (-0.117,0.012); M 85+ (-0.083,0.009)  F < 45 (-0.844,0.086); F 45-54 (-0.734,0.075)  F 55-64 (-0.431,0.044); F 65-74 (-0.261,0.027)  F 75-84 (-0.174,0.018); F 85+ (-0.051,0.005) | Prospective studies collaborative meta-analysis (2007) [54]. Parameters on the log-scale. |
| Aspirin  Beta blockers  ACE Inhibitors  Statins  Rehabilitation  Warfarin | M & F (0.15,0.139)  M & F (0.23,0.185)  M & F (0.20,0.177)  M & F (0.24,0.245)  M & F (0.26,0.347)  M & F (0.22,0.305) | ATC (2002) [35]  Freemantle (1999) [29]  Flather (2000) [40]  Hulten (2006) [41]  Taylor (2004) [43]  Anand and Yusuf (1999) [42] |
| **Primary prevention therapies: Statins** | | |
| **Eligible patients**:  Population | Population counts (no error) |  |
| **Treatment uptake** | % never having had angina or heart attack and currently taking lipid lowering drugs prescribed by a doctor: (***Beta distribution***: cases, sample-size minus cases) |  |
| **Case fatality rate** | Sample size (*n*) = never having had angina or heart attack and currently taking lipid lowering drugs in 2006:  ***Beta distribution*** (cases = *n* × CFR estimate, non-cases = *n* – cases) | Wijeysundera et al (2010) [5] |
| **Compliance** | ***Beta distribution*** (cases = *n* × assumed compliance, non-cases = *n* – cases) | Wijeysundera et al (2010) [5] |
| **Relative risk reduction:** Statins | ***Ersatz RR function*** (RRR, SE ln(RRR)):  M & F (0.35,0.396) | Pignone (2000) [52] |
| **Primary prevention therapies: Treatments for high blood pressure** | | |
| **Eligible patients**:  Population | Population counts (no error) |  |
| **Treatment uptake** | % never having had angina or heart attack and currently taking medication specifically prescribed to treat high blood pressure: (***Beta distribution***: cases, sample-size minus cases) |  |
| **Case fatality rate** | Sample size (*n*) = never having had angina or heart attack and currently taking medication to lower blood pressure in 2006:  ***Beta distribution*** (cases = *n* × CFR estimate, non-cases = *n* – cases) | Wijeysundera et al (2010) [5] |
| **Compliance** | ***Beta distribution*** (cases = *n* × assumed compliance, non-cases = *n* – cases) |  |
| **Relative risk reduction:** Treatments for high blood pressure | ***Ersatz RR function*** (RRR, SE ln(RRR)):  M & F (0.13,0.294) | Law (2003) [51] |
